# Documentation of guideline adherence in antenatal records across maternal weight categories: a chart review

**DOI:** 10.1186/1471-2393-14-205

**Published:** 2014-06-13

**Authors:** Sarah D McDonald, Clea A Machold, Laura Marshall, Dawn Kingston

**Affiliations:** 1 Division of Maternal-Fetal Medicine, Departments of Obstetrics & Gynecology and Diagnostic Imaging and Clinical Epidemiology & Biostatistics, McMaster University, 1280 Main St. West, HSC 3N52B, Hamilton, ON L8S4K1, Canada; 2 Department of Obstetrics, Gynecology1280 Main St. West, HSC 3 N52, Hamilton, ON L8S4K1, Canada; 3 Faculty of Health Sciences, Department of Obstetrics, Gynecology1280 Main St. West, HSC 3 N52, Hamilton, ON L8S4K1, Canada; 4 Level 3, Edmonton Clinic Health Academy, University of Alberta, 11405 87 Avenue, Edmonton, Alberta T6G 1C9, Canada

**Keywords:** Antenatal medical records, Documentation, Guideline adherence, Obesity, Prenatal care

## Abstract

**Background:**

Documentation in medical records fulfills key functions, including management of care, communication, quality assurance and record keeping. We sought to describe: 1) rates of standard prenatal care as documented in medical charts, and given the higher risks with excess weight, whether this documentation varied among normal weight, overweight and obese women; and 2) adherence to obesity guidelines for obese women as documented in the chart.

**Methods:**

We conducted a chart review of 300 consecutive charts of women who delivered a live singleton at an academic tertiary centre from January to March 2012, computing Analysis of Variance and Chi Square tests.

**Results:**

The proportion of completed fields on the mandatory antenatal forms varied from 100% (maternal age) to 52.7% (pre-pregnancy body mass index). Generally, documentation of care was similar across all weight categories for maternal and prenatal genetic screening tests, ranging from 54.0% (documentation of gonorrhea/chlamydia tests) to 85.0% (documentation of anatomy scan). Documentation of education topics varied widely, from fetal movement in almost all charts across all weight categories but discussion of preterm labour in only 20.6%, 12.7% and 13.4% of normal weight, overweight and obese women’s charts (p = 0.224). Across all weight categories, documentation of discussion of exercise, breastfeeding and pain management occurred in less than a fifth of charts.

**Conclusion:**

Despite a predominance of excess weight in our region, as well as increasing perinatal risks with increasing maternal weight, weight-related issues and other elements of prenatal care were suboptimally documented across all maternal weight categories, despite an obesity guideline.

## Background

Obesity during pregnancy is now highly prevalent, and is recognized by care providers as an important health issue [1] due to associated complications. Obesity affects at least 16-19% of pregnant women in the United Kingdom [2], and 11% [3] - 40% [4] of pregnant women in America, with overweight affecting an additional 12% [5] -38% [4]. Obese women are at increased risks of both maternal complications (including excess weight gain and gestational diabetes) [6] and fetal complications (such as anomalies including neural tube defects and preterm birth) [7].

The increased risk of complications in obese women is particularly concerning as outside of pregnancy obese women have been found to be less likely to seek or receive key elements of health care including screening [8-10].

To try to facilitate high quality health care, standardized Antenatal Record forms are mandatory in many jurisdictions including in the province of Ontario, where this research was conducted [11]. Additionally, recommendations for care of obese women have been summarized in national guidelines by multiple organizations, including the Centre for Maternal and Child Enquiries jointly with the Royal College of Obstetricians and Gynaecologists (CMACE/RCOG), the American College of Obstetrics and Gynecology (ACOG) and the Society of Obstetricians and Gynecologists of Canada (SOGC) [2,12,13]. We sought to determine documentation of adherence to recommendations for standard prenatal care for women across all weight classes, and given the higher perinatal risks in women of excess weight, whether this documentation varied among normal weight, overweight and obese women. Our secondary objective, in the case of obese women, was to determine the degree of documented adherence to obesity guidelines [12,13].

## Methods

We conducted a chart review. We included all women who gave birth to a live singleton infant at an academic tertiary care centre, McMaster University Medical Centre in Hamilton, Ontario, Canada between January 2012 and March 2012 and whose charts included the Ministry of Health mandated Antenatal Records. We reviewed 300 consecutive charts meeting inclusion criteria as a convenience sample.

Our research questions were: (1a) What proportion of charts have documentation of standard prenatal care? 1b) Compared to women with normal weight, do overweight or obese women have similar documentation of standard prenatal care; and (2) In the case of obese women, does their documented antenatal care reflect the specific recommendations related to weight in the guideline on obesity during pregnancy [13]?

We hypothesized that documentation would be suboptimal across all weight categories in terms of standard prenatal care but that certain types of screening, such as Papanicolaou tests and gonorrhea and chlamydia tests might not be as frequent in obese women due to technical difficulties. Secondly, we hypothesized that obese women would have more complete documentation of weight-related issues, reflecting the guideline on obesity during pregnancy [13].

With regards to outcomes related to standard prenatal care, we examined the documentation of the following components in the antenatal record:

i. *maternal screening*: pre-pregnancy weight, height and BMI calculation, weight at subsequent visits, Papanicolaou test, gonorrhea and chlamydia tests, and gestational diabetes, and Group Beta Streptococcus (GBS) status;

ii. *prenatal genetic screening*: the Integrated Prenatal Screen (IPS), First Trimester Screening (FTS) or Maternal Serum Screening (MSS) or chorionic villus sampling or amniocentesis, and first trimester [14] and second trimester ultrasound scans, and

iii. *counseling and education* on 21 ‘discussion topics’ addressing subjects such as exercise, fetal movement, preterm labour, prenatal classes, birth plans, pain management, and breastfeeding.

With regards to outcomes related to prenatal care specific to obese women, we examined the following components of documentation in the Antenatal Record of the prenatal care portion of the obesity guidelines [13]:

i. body mass index (BMI) - calculated from pre-pregnancy height and weight;

ii. counseling about weight gain, nutrition, and food choices;

iii. counseling about increased risk of congenital abnormalities, and appropriate screening;

iv. counseling about increased risk for medical complications (such as gestational diabetes) and that regular exercise during pregnancy may help to reduce some of these risks.

We focused on the prenatal care portion of the SOGC guidelines on obesity, [13] which are very similar to the CMACE/RCOG guidelines [2] and the ACOG guidelines [12] as we were interested in prenatal care (not intrapartum care). Additionally, many of these items already appear on the Ministry of Health-mandated Antenatal Records and hence had the highest likelihood of being documented. All four of the topics that the guideline [13] emphasized for the prenatal care of obese women (calculation of pre-pregnancy BMI, counseling about weight gain, counseling about complications such as gestational diabetes, and counseling about congenital anomalies) are also part of standard prenatal care for all weight classes of women.

As per the Institute of Medicine Guidelines (also adopted by Health Canada), [15,16] we classified women according to the World Health Organization pre-pregnancy body mass index (BMI) weight categories (underweight: BMI <18.5 kg/m^2^, normal weight: BMI 18.5-24.9 kg/m^2^, overweight: BMI 25.0-29.9 kg/m^2^, and obese: BMI ≥30 kg/m^2^). If the care provider had not calculated BMI, then we calculated it based on the height reported in the chart. When the height was not recorded, we used weight alone as in previous studies to categorize women as overweight [17] (70–89 kg) and obese [13,17,18] (≥90 kg).

We reviewed the hospital charts of the patients, consisting of both information gathered in hospital as well as the care providers’ office charts which are faxed to the hospital typically in the later third trimester. Health care providers in Ontario record antenatal information on government-mandated Ministry of Health forms called Antenatal Record 1 and 2, which contain similar fields to the National Institute for Health Care Excellence (NICE) antenatal care pathways [19] and the ACOG Antepartum Record [20]. The first page of the Antenatal Record contains information on demographics of the mother and partner including occupation, education and marital status, the dating of the pregnancy, medical history and physical exam, initial laboratory investigations, and prenatal genetic investigations.

The second page of the Antenatal Record contains identified risk factors, information on each visit (including the date and number of weeks gestation, weight, blood pressure, proteinuria, fetal heart rate, and comments), ultrasound results, additional Lab Investigations including Glucose Challenge Test (GCT), Glucose Tolerance Test (GTT), plan of management and a list of 21 discussion topics. The 21 discussion topics are generally addressed throughout the course of the latter half of the pregnancy. Results from any tests are to be recorded on the mandatory Antenatal Records.

For all outcomes, we reviewed the Antenatal Records as well as the entire hospital chart (including the initial referral letter and discharge summary). A trained research assistant used a standardized data collection form to abstract data. The Hamilton Health Sciences/McMaster University Faculty of Health Sciences Research Ethics Board approved the study prior to its initiation (REB Project # 12-315-C).

We first generated descriptive statistics (proportions, means and standard deviations) of the sample characteristics and outcomes. To determine if differences in the outcomes were evident across our 3 groups of interest - women with normal weight, overweight and obesity - we used Analysis of Variance (ANOVA) for continuous variables and Chi Square tests for proportions. In accordance with our multiple outcomes, statistical significance was set at p < 0.001. In a post hoc exploratory analysis, we examined whether the degree of documentation varied by the type of care provider, since midwifery visits generally last longer [21-23].

This study was approved by the Hamilton Health Sciences/McMaster University Faculty of Health Sciences Research Ethics Board, Project # 12-315-C, June 21, 2012.

## Results

In our audit of 300 consecutive charts of women who gave birth at our academic tertiary care centre over a 3-month period (Table 1), the proportion of completed fields on the Ministry of Health mandated antenatal forms varied from 100% for maternal age to 53% for BMI. Descriptive statistics are shown in Table 1. The mean maternal age at the time of first visit was 29.9 years (SD 5.8), the majority of women were Caucasian (58%), either married or in a common-law relationship (77%) and worked outside the home (60%).

**Table 1:** Baseline characteristics of the study sample

**Variable**	**Number and proportion (%) of study sample unless otherwise indicated**
**Maternal age**	
Years, mean (SD)	29.9 (5.8)
< 35 years	231 (77.0%)
≥ 35 years	69 (23.0%)
**Ethnicity**	
Caucasian	174 (58.0%)
Other	64 (21.3%)
Blank	59 (19.7%)
Not legible	3 (1.0%)
**Marital status**	
Married or common-law	230 (76.7%)
Single	36 (12.0%)
Blank	33 (11.0%)
Unclear (not legible or discrepant documentation)	1 (0.3%)
**Education**	
Post-secondary	156 (52.0%)
Secondary or less	57 (19.0%)
Blank	83 (27.7%)
Not legible	4 (1.3%)
**Occupation**	
Work outside the home	180 (60.0%)
Homemaker	50 (16.7%)
Student	17 (5.7%)
Unemployed	19 (6.3%)
Blank	24 (8.0%)
Unclear (not legible or discrepant documentation)	10 (3.3%)
**Currently smoking**	44 (14.7%)
**Pregnancy history**	
First time giving birth	86 (28.7%)
One or more previous births	214 (71.3%)
**BMI classification**	
Underweight (BMI ≤ 18.4 kg/m^2^)	11 (3.7%)
Normal weight (BMI 18.5-24.9 kg/m^2^)	126 (42.0%)
Overweight (BMI 25.0-29.9 kg/m^2^) or when height unavailable weight of 70–89 kg [17] (most pre-pregnancy)	79 (26.3%)
Obese (BMI ≥ 30 kg/m^2^) or when height unavailable weight of ≥ 90 kg [13,17,18] (most pre-pregnancy)	82 (27.3%)
No weight recorded anywhere on chart	2 (0.7%)

Just over half of women had BMI recorded by the care provider (53%) in the Antenatal Record, while 40% had pre-pregnancy height and weight recorded without BMI being calculated. Additionally, there were 19 women for whom height was not recorded. Among these 19 women, weight was recorded for 8 in the first trimester, 7 in the second trimester, and 4 in the third trimester while for 2 there was no weight available anywhere in the chart. According to pre-pregnancy BMI, 42% were normal weight, 26% were overweight 27% were obese, while only 4% were underweight.

Outcomes for the whole study population are shown in Table 2, and additionally are compared by weight category. Documentation of antenatal care components varied by outcome, with almost all women having weight recorded at every visit (92%), but only 54% of charts having documentation of swabbing for gonorrhea and chlamydia. Overall, documentation of care was similar amongst normal weight, overweight and obese women in terms of weight recorded prenatally and at antenatal visits, performance of Papanicolau testing, gonorrhea and chlamydia screening, first ultrasound performed during the first trimester (as per another SOGC guideline) [14] , prenatal genetic screening, swabbing for group B streptococcus and education topics .

**Table 2:** Outcomes in whole study sample

**Variable**	**Number and proportion (%) of study sample**	**Number and proportion (%) for normal weight (BMI = 18.5-24.9)**	**Number and proportion (%) for overweight (BMI = 25.0-29.9 kg/m**^**2**^**)**	**Number and proportion (%) for obese (BMI ≥ 30 kg/m**^**2**^**)**	**P value**
	N = 300^1^	N = 126	N = 79	N = 82	N/A
**Pre-pregnancy BMI recorded in chart**^2^	159 (53.0%)	71 (56.3%)	42 (53.2%)	39 (47.6%)	0.71
**Weight recorded at antenatal visits**					0.88
Always	277 (92.3%)	115 (91.3%)	74 (93.7%)	77 (93.9%)	
Never	4 (1.3%)	1 (0.8%)	0 (0.0%)	1 (1.2%)	
Sometimes	19 (6.3%)	10 (7.9%)	5 (6.3%)	4 (4.9%)	
**Documented recommendation of weight gain**^2^	41 (13.7%)	20 (13.7%)	7 (8.9%)	14 (17.1%)	0.26
**Obesity correctly identified a risk factor**^8^	76/82 (92.7%)	0 (0.0%)	4 (5.1%)	76 (92.7%)	N/A
**Papanicolaou test**					0.87
Documented within last year	197 (65.7%)	84 (66.7%)	53 (67.1%)	52 (63.4%)	
Documented greater than 1 year ago	28 (9.3%)	10 (7.9%)	6 (7.6%)	11 (13.4%)	
Not documented anywhere in chart	54 (18.0%)	24 (19.0%)	14 (17.7%)	13 (15.9%)	
Date not legible	21 (7.0%)	8 (6.3%)	6 (7.6%)	6 (7.3%)	
**Gonorrhea and chlamydia tests**					0.67
Documented as swabbed	162 (54.0%)	70 (55.6%)	44 (55.7%)	42 (51.2%)	
Documented as not swabbed	6 (2.0%)	4 (3.2%)	0 (0.0%)	2 (2.4%)	
Not documented anywhere in chart	131 (43.7%)	51 (40.5%)	35 (44.3%)	38 (46.3%)	
Not legible	1 (0.3%)	1 (0.8%)	0 (0.0%)	0 (0.0%)	
**Prenatal genetic screening**^3^					0.37
Documented accepted^5^	153 (51.0%)	68 (54.0%)	32 (40.5%)	46 (56.1%)	
Documented declined or too late for tests	90 (30.0%)	35 (27.8%)	31 (39.2%)	21 (25.6%)	
No results or offer documented anywhere in chart	35 (11.7%)	13 (10.3%)	10 (12.7%)	11 (13.4%)	
Unclear (documented as both accepted and declined or too late^4^)	22 (7.3%)	10 (7.9%)	6 (7.6%)	4 (4.9%)	
**First ultrasound scan**					
Documented in first trimester	212 (70.7%)	89 (70.6%)	54 (68.3%)	60 (73.2%)	0.09
**Second trimester 18–22 week ultrasound scan for anatomy**^2^	255 (85%)	109 (86.5%)	69 (87.3%)	65 (79.3%)	0.27
No ultrasounds documented anywhere in chart	8 (2.7%)	5 (4.0%)	0 (0.0%)	2 (2.4%)	
Unclear (dates not legible or discrepant documentation^5^)	1 (0.3%)	1 (0.8%)	0 (0.0%)	0 (0.0%)	
**Gestational diabetes testing**^2^ (Glucose challenge test and/or Glucose tolerance test)					0.09
Documented as completed	207 (69.0%)	90 (71.4%)	60 (75.9%)	51 (62.2%)	
Documented as not completed	28 (9.3%)	15 (11.9%)	6 (7.6%)	6 (7.3%)	
Not documented anywhere in chart	65 (21.7%)	21 (16.7%)	13 (16.5%)	25 (30.5%)	
**Group B streptococcus screening**					0.05
Documented as swabbed^6^	259 (86.3%)	109 (86.5%)	69 (87.3%)	71 (86.6%)	
Documented as not swabbed^7^	21 (7.0%)	13 (10.3%)	6 (7.6%)	2 (2.4%)	
Not documented anywhere in chart	20 (6.7%)	4 (3.2%)	4 (5.1%)	9 (11.0%)	
**Primary health care provider during pregnancy**					0.01
Shared antenatal care^9^	125 (41.7%)	51 (40.5%)	42 (53.2%)	28 (34.1%)	
Exclusive care by Obstetrician	121 (40.3%)	46 (36.5%)	24 (30.4%)	45 (54.9%)	
Exclusive care by Midwife	53 (17.7%)	28 (22.2%)	13 (16.5%)	9 (11.0%)	
Exclusive care by Family Physician	1 (0.3%)	1 (0.8%)	0 (0.0%)	0 (0.0%)	
**Counseling and education topics**					
Discussion of exercise checked off or written in chart^2^	40 (13.3%)	17 (13.5%)	14 (17.7%)	6 (7.3%)	0.14
Discussion of preterm labor checked off or written in chart	50 (16.7%)	26 (20.6%)	10 (12.7%)	11 (13.4%)	0.22
Discussion of fetal movement checked off or written in chart	293 (97.7%)	123 (97.6%)	78 (98.7%)	80 (97.6%)	1.00
Discussion of prenatal classes checked off or written in chart	41 (13.7%)	20 (15.9%)	12 (15.2%)	7 (8.5%)	0.28
Discussion of birth plans checked off or written in chart	116 (38.7%)	47 (37.3%)	31 (39.2%)	37 (45.1%)	0.52
Discussion of pain management checked off or written in chart	32 (10.7%)	15 (11.9%)	6 (7.6%)	8 (9.8%)	0.60
Discussion of breastfeeding checked off or written in chart	33 (11.0%)	16 (12.7%)	6 (7.6%)	10 (12.2%)	0.50

^1^No available BMI or weight for 2/300 women who were excluded from these analyses; 11/300 underweight were excluded from these analyses due to the small number; therefore, total included in these analyses N = 287.

^2^Indicates a recommendation within the obesity guidelines, while the remainder of the outcomes are part of standard antenatal care for all women regardless of weight category, as noted on the mandatory antenatal records (with regard to gestational diabetes, the guideline recommends advising obese women that they are at risk for medical complications including gestational diabetes).

^3^Integrated prenatal screen (IPS), or maternal serum screen (MSS) or Chorionic villus sampling/amniocentesis for Down Syndrome.

^4^Documented as both completed and declined or too late on first page of antenatal record, but no documentation confirming decision elsewhere in chart.

^5^Documented as scheduled in visit notes on second page of antenatal record, but no documentation confirming completion elsewhere in chart.

^6^“Positive” or “Negative” for Group B streptococcus or “Yes” documented for “Group B streptococcus at 35–37 weeks?” in maternal record.

^7^“No” documented for “Group B streptococcus at 35–37 weeks?” in maternal record.

^8^Obesity was incorrectly identified as a risk factor in 4/79 overweight women.

^9^Any combination of Obstetrician, Maternal-Fetal Medicine, Family Physician, Midwife and/or Nurse Practitioner.

The extent to which education topics were documented as being covered varied widely by topic. For instance, while education on fetal movement was documented in almost all charts, discussion of preterm labour was documented in only 20.6%, 12.7% and 13.4% of normal weight, overweight and obese women’s charts (p = 0.22). Although a discussion of a birth plan was documented in more than a third of charts, less than a fifth of charts documented discussions related to prenatal classes, breastfeeding, and pain management.

Less than 20% of women across all weight categories had documentation of a recommendation for a particular amount of weight gain (13.7%, 8.9%, and 17.1% of normal weight, overweight, and obese women, respectively, p = 0.26).

The majority of women had documentation of a second trimester ultrasound performed for anatomical assessment between 18–22 weeks, with 86.5%, 87.3% and 79.3% of normal weight, overweight and obese women’s charts noting this (p = 0.27) .

Documentation of screening for gestational diabetes was suboptimal, occurring in 71%, 76% and 62% of normal weight, overweight, and obese women’s charts (p = 0.086).

Exercise was rarely checked off in the discussion topics or written anywhere in the chart (occurring in only 13.5%, 17.7%, and 7.3% of normal weight, overweight, and obese women’s charts, p = 0.14).

In a post hoc exploratory analysis, we noted that charts of women under exclusive midwifery care generally had higher proportions of documentation of discussion topics, but also had higher rates of documentation indicating that swabbing for gonorrhea or chlamydia was not done, and that genetic screening was declined or too late to be offered to the woman (data not shown).

## Discussion

Documentation in prenatal records fulfills key functions, including patient care management, communication, quality assurance and record keeping for legal purposes [24]. We found suboptimal documentation in many areas of prenatal care and the observed rates of documentation did not increase across weight categories despite both increasing perinatal risks, and guidelines specifically addressing recommendations related to weight in obese women [12,13].

Just over half of all women had documentation of pre-pregnancy BMI, despite the fact that the government mandated Antenatal Records contain a space specifically for it, and it is a specific recommendation of the obesity guidelines that it be calculated [12,13]. Moreover, prenatal weight gain guidelines are based on pre-pregnancy BMI [15,16] and hence cannot be followed accurately without knowledge of pre-pregnancy BMI. Additionally, counseling about weight gain is an explicit recommendation in the obesity guidelines [12,13] although we found that only a minority of charts either overall or in obese women contained documentation of a recommendation of weight gain. Low rates are concerning given that a lack of counseling about weight gain is associated with inappropriate gestational weight gain, both inadequate and excessive, compared to having received counseling by a health care provider [25]. We are not aware of other studies that examined documentation of adherence to guidelines according to weight category, apart from a single study that examined only gestational weight gain and not the additional outcomes. This study, located in a Massachusetts tertiary care center, found even lower rates (only 4.6%) of documentation of pre-pregnancy BMI in the antenatal records [26].

Previous work has shown that the creation and dissemination of a guideline does not equate with implementation [27]. For instance, only 50% of Australian antenatal care providers correctly identified the BMI categories and only 32% were aware of the weight guidelines [1]. Barriers to adherence to guidelines include lack of awareness of guidelines, lack of motivation, lack of time and lack of outcome expectancy (belief that the desired outcome will be achieved) [28,29]. Strategies for overcoming barriers to knowledge translation at an individual practitioner level, audit and feedback, reminders and multifaceted strategies have been shown to be particularly effective in obstetrics literature [30]. At a system level include: ensuring liberal access to internet facilities including databases tailored to each particular specialty at point of care (if the barrier consisted of poor access to best evidence), and at a local workplace level of having regular evidence-based rounds and mentors including change mentors, innovators and educators (if the barrier was lack of knowledge) [31]. However, in our study, the issue is unlikely to be one of inadequate knowledge translation concerning the guideline, given that in previous research [32], the same practitioners as those whose charts were included in this study, generally demonstrated awareness of the guideline by recommending the correct amount of weight gain for pregnant women [32].

Beyond knowledge translation, to address the suboptimal rates of documentation that we found in this study, there are a number of diverse, innovative approaches which could be employed, including use of electronic medical records, and patient involvement. Electronic medical records could, for instance, automatically calculate BMI after height and weight were entered, and could prompt care providers to discuss appropriate weight gain. Such automated, computerized reminders been found to be effective for improving consistency of routine practices [33]. The potential beneftis of electronic medical records, particularly in obstetrics, has been emphasized: “Obstetrics is an ideal speciality for both the implementation and evaluation of the effectiveness of a paperless record. It is a well-defined field with a relatively standard course of care for a common condition, although is challenged by the diversity of providers (e.g., obstetricians, midwives, family physicians, nurses). Communication among providers is a key element in ensuring quality care, because all patients have their care transferred from outpatient to inpatient settings and back and between different providers, and patients may be cared for by different providers” [34]. Alternately, since much of obstetrical care involves young, healthy women, engaging them to a much larger extent in their care including self-documentation in their charts, is a realistic potential step. Indeed, this approach has been effectively utilized in group prenatal care models in which women describe their preference for sharing ownership of care through increased involvement in weighing themselves, taking their own blood pressure, and charting in their medical record [35]. Self-documentation may have other benefits, such as more complete data than is provided by health care workers, as has been noted in the case of some aspects of care documented by parents whose newborns were admitted to an intensive care unit [36].

Appropriate documentation is an important feature of quality health care, [37] managing risk and medical liability [38]. It has been advocated that “there must be a disciplined approach to documentation” [38]. Risk management recommendations include thorough documentation of the past obstetrics history for instance, for pediatricians examining neurologic impairment in newborns [39] and it would be even more important to have complete information about past obstetrics history in obstetric care providers’ charts. Improvement of documentation might occur through medical informatics, which The Institute of Medicine’s report *To Err is Human*[40] suggested might decrease medical errors and enhance patient safety.

A main strength of our study is that we did not rely solely on the Antenatal Record Forms, but used other sources including dictated consultations and physician notes, allowing us to decrease the amount of missing data. A single trained data abstractor performed all of the chart reviews, and while we did not perform double-checking, this eliminated variation between abstractors. We chose to study variables that were standard of care and/or guideline driven. We deliberately included various types of care providers, including obstetricians, midwives and family physicians to obtain a broad sample of care providers whose time constraints vary widely.

Limitations of the study include the fact that our observations were limited to a single academic tertiary care centre. Within this centre there are both high and low risk births, cared for by all types of antenatal care providers being midwives, family physicians, obstetricians. Previous work has found better documentation in high risk charts [41]. Our study took place at a tertiary care centre that provides care to both low and high risk women; however, despite the fact that obese women have a higher risk of complications including preterm birth, documentation was not better among obese women in our study. We reviewed the hospital charts of the patients, and it is possible that there was additional information in the care providers’ office charts although typically all of the contents are faxed to the hospital chart. Another limitation is that our sample size of 300, particularly when broken down into the three weight groups, resulted in some outcomes with small numbers. This may have resulted in false negative associations between some weight categories and some outcomes.

Future research should explore ways of improving documentation, including the impact of electronic documentation, and plans are under way to implement this in our hospital clinics in the near future. Following audit, feedback is an important step to improving care, [42] and will be our next goal, beginning with grand rounds in our hospital. Further education of care providers about the importance of documentation in general and about the content of guidelines, such as the weight gain guidelines, will be important.

## Conclusions

In conclusion, despite a predominance of excess weight in our region, weight-related issues and other important healthcare issues, were suboptimally documented in the chart. Rates of documentation did not increase across weight categories despite both increasing perinatal risks, and guidelines specifically addressing recommendations related to weight in obese women. These finding are important as there is a need to improve documentation, both for optimal care management, communication and quality assurance, but also for the relevance of record keeping for legal cases as the “silent witness” [38].

## Abbreviations

ACOG: American College of Obstetrics & Gynecology; SOGC: Society of Obstetricians & Gynecologists of Canada; BMI: Body Mass Index; CMACE/RCOG: Centre for Maternal and Child Enquiries/Royal College of Obstetricians and Gynaecologists; GBS: Group Beta Steltococcus status; IPS: Integrated prenatal screen; FTS: First trimester screen; MSS: Maternal serum screen; GCT: Glucose challenge test; GTTL: Glucose tolerance test; ANOVA: Analysis of variance; SD: Standard deviation.

## Competing interests

The authors declare that they have no competing interests’.

## Authors’ contributions

SDM and DK conceived the study and designed it, CM and LM participated in the design of the study and performed chart data abstraction, SDM drafted the manuscript and edited it with DK, and all authors approved the final version of the manuscript.

## Authors’ information

Dr Sarah McDonald (MD, MSc in Clinical Epidemiology) is a Maternal-Fetal Medicine specialist (high risk obstetrician) and Associate Professor in the Department of Obstetrics and Gynecology at McMaster University. As a Clinician-Scientist her salary is supported by a Canadian Institute of Health Research New Investigator Award. Her research in perinatal epidemiology focuses on weight-related issues during pregnancy and the postpartum period, and on related health services. Dr Dawn Kingston, RN, PhD, is an Assistant Professor in the Faculty of Nursing University of Alberta. Her research program focuses on antenatal and postnatal depression and anxiety, and addressing their health services issues.

## Pre-publication history

The pre-publication history for this paper can be accessed here:

http://www.biomedcentral.com/1471-2393/14/205/prepub
